# 5-HT1A receptor-dependent modulation of emotional and neurogenic deficits elicited by prolonged consumption of alcohol

**DOI:** 10.1038/s41598-018-20504-z

**Published:** 2018-02-01

**Authors:** Arnauld Belmer, Omkar L. Patkar, Vanessa Lanoue, Selena E. Bartlett

**Affiliations:** 1 Translational Research Institute, Queensland University of Technology, Brisbane, 4100 Australia; 2 Institute of Health and Biomedical Innovation (IHBI), Queensland University of Technology, 4100 Brisbane, Australia; 3 Clem Jones Centre for Ageing Dementia Research (CJCADR), Queensland Brain Institute, University of Queensland, Brisbane, 4100 Australia

## Abstract

Repeated episodes of binge-like alcohol consumption produce anxiety, depression and various deleterious effects including alterations in neurogenesis. While the involvement of the serotonin receptor 1 A (5-HT_1A_) in the regulation of anxiety-like behavior and neurogenesis is well documented, its contribution to alcohol withdrawal-induced anxiety and alcohol-induced deficits in neurogenesis is less documented. Using the Drinking-In-the-Dark (DID) paradigm to model chronic long-term (12 weeks) binge-like voluntary alcohol consumption in mice, we show that the selective partial activation of 5-HT_1A_ receptors by tandospirone (3 mg/kg) prevents alcohol withdrawal-induced anxiety in a battery of behavioral tests (marble burying, elevated-plus-maze, open-field), which is accompanied by a robust decrease in binge-like ethanol intake (1 and 3 mg/kg). Furthermore, using triple immunolabelling of proliferation and neuronal differentiation markers, we show that long-term DID elicits profound deficits in neurogenesis and neuronal fate specification in the dorsal hippocampus that are entirely reversed by a 2-week chronic treatment with the 5-HT_1A_ partial agonist tandospirone (3 mg/kg/day). Together, our results confirm previous observations that 5-HT_1A_ receptors play a pivotal role in alcohol drinking behavior and the associated emotional and neurogenic impairments, and suggest that 5-HT_1A_ partial agonists represent a promising treatment strategy for alcohol abuse.

## Introduction

Alcoholism is regarded as a chronic relapsing disorder, with the development of alcohol addiction a progressive cycle involving extended periods of heavy alcohol use, with repeated episodes of binge-like consumption and abstinence^1^. In society, alcohol abuse is highly prevalent with significantly higher rates of co-occurrence with emotional and mood disorders including anxiety and depression^2^. In turn, increased anxiety levels following alcohol withdrawal are key factors contributing to craving and relapse^3^. Animal studies using non-contingent/forced alcohol delivery (e.g., vapor inhalation, injection, gavage) or contingent models based on continuous access to alcohol (2-bottle choice) have also revealed that withdrawal from chronic alcohol exposure increases stress responsiveness^4^, anxiety-^5^ and depression-like behaviors^6^, and alters hippocampal neurogenesis^7^. However, no study to date has investigated the consequences of prolonged periods (12 weeks) of voluntary binge-like alcohol consumption and abstinence on anxiety-like behaviors and hippocampal neuron proliferation or differentiation.

The “Drinking-In-the-Dark” (DID) paradigm is a model of voluntary binge-like alcohol drinking, in which animals have a daily 2-hour limited access to ethanol followed by a 24-hour abstinence period. Using this procedure, the high-drinking C57Bl/6 J mouse strain consumes pharmacologically relevant levels of ethanol (blood ethanol concentration ≥1.0 g/L) and shows signs of behavioral intoxication^8^. In adult mice chronically exposed for short term periods (6 weeks), this procedure was shown to elicit increased anxiety- and depression-like behaviors as early as 24 hours after the last drinking session and up to 21 days into protracted withdrawal^9^.

The serotonin (5-hydroxytryptamine, 5-HT) system has been widely implicated in the regulation of emotion, impulsivity, mood, reward and arousal. As such, dysregulation of serotonin homeostasis is distinctly involved in the development of anxiety- and depression-related disorders. In line with this, profound functional changes in 5-HT signaling have been observed in the limbic system following acute and chronic alcohol exposure^10^. Since selective serotonin reuptake inhibitor (SSRI) antidepressants elevate 5-HT availability and promote hippocampal neurogenesis, which is required for antidepressant effects^11^, a positive relationship between 5-HT signaling and hippocampal neurogenesis has been established, with the role of the 5-HT_1A_ receptor becoming well elucidated in both anxiety/depression^12,13^ and neurogenesis^14^.

Chronic alcohol exposure downregulates 5-HT_1A_ receptor expression in the hippocampus but upregulates 5-HT_1A_ receptor expression and function in the raphé^15,16^. Interestingly, 5-HT_1A_ receptor activation by the non-selective partial agonists buspirone was shown to reduce alcohol drinking^17^ and alcohol withdrawal-induced anxiety^18–20^ in rodents. These particular studies however, used a protocol in which animals are forced to consume alcohol by including ethanol in a liquid diet as the sole source of nutrient, making their motivation to drink ethanol in relation to emotional deficits questionable. Additionally, it is now well accepted that buspirone is not selective for 5-HT_1A_ receptors but also displays an antagonist activity at dopamine D_2_-like receptors^21,22^. Indeed, the effect of buspirone on alcohol drinking as well as some anxiety-related behaviors are mediated by D2-like receptor blockade^23,24^. Furthermore, we and others have demonstrated that buspirone has a complex action with both anxiolytic and anxiogenic, or motor effects in rodents^25–30^, which could explain the inconsistent results on the benefit of buspirone reported in human alcoholics^31^. Together, these data suggest that that further work is needed to clarify the specific contribution of 5-HT_1A_ receptors in both alcohol drinking behaviour and alcohol-withdrawal induced anxiety.

Tandospirone is a 5-HT_1A_ partial agonist that, unlike buspirone, presents a high selectivity of two to three orders of magnitude over dopamine, adrenergic and other 5-HT receptors^32^. Tandospirone is an effective anxiolytic drug marketed as Sediel in China and Japan, that is well tolerated and presents limited adverse effects and low abuse liability^33^. Animal studies have also demonstrated that chronic tandospirone treatment improves hippocampal neurogenesis^34^ and inhibits stress-induced anxiety and neurogenic deficits^35^. However, the ability of tandospirone to reduce anxiety-like behavior and neurogenic deficits following long-term binge-like alcohol consumption has not been described. To address this question, we used the long-term DID (12 to 15 weeks) model to investigate the effect of tandospirone on (1) alcohol withdrawal-induced anxiety in a battery of behavioral tests (marble burying, elevated-plus-maze and open-field), (2) the maintenance of alcohol binge-like drinking and (3) alcohol-induced deficits in hippocampal neurogenesis. Here, we report for the first time that the selective partial activation of 5-HT_1A_ receptors by tandospirone reverses alcohol withdrawal-induced anxiety-like behaviors following 12 weeks of exposure, which is accompanied by a robust decrease in alcohol binge-like consumption. In addition, we show that a 2-week tandospirone treatment is sufficient to totally reverse the impairments in hippocampal neurogenesis and neuronal fate specification caused by 15 weeks of binge-like alcohol consumption. Taken together, our results complement previous studies by demonstrating the the integral role of 5-HT_1A_ receptors in emotion-driven alcohol binge-like drinking behavior and neurogenic impairments elicited by prolonged binge-like consumption of alcohol.

## Results

### Partial activation of 5-HT_1A_ receptors by tandospirone reverses anxiety-like behavior elicited by withdrawal from 12 weeks of binge-like alcohol consumption in the DID

**Figure 1:**
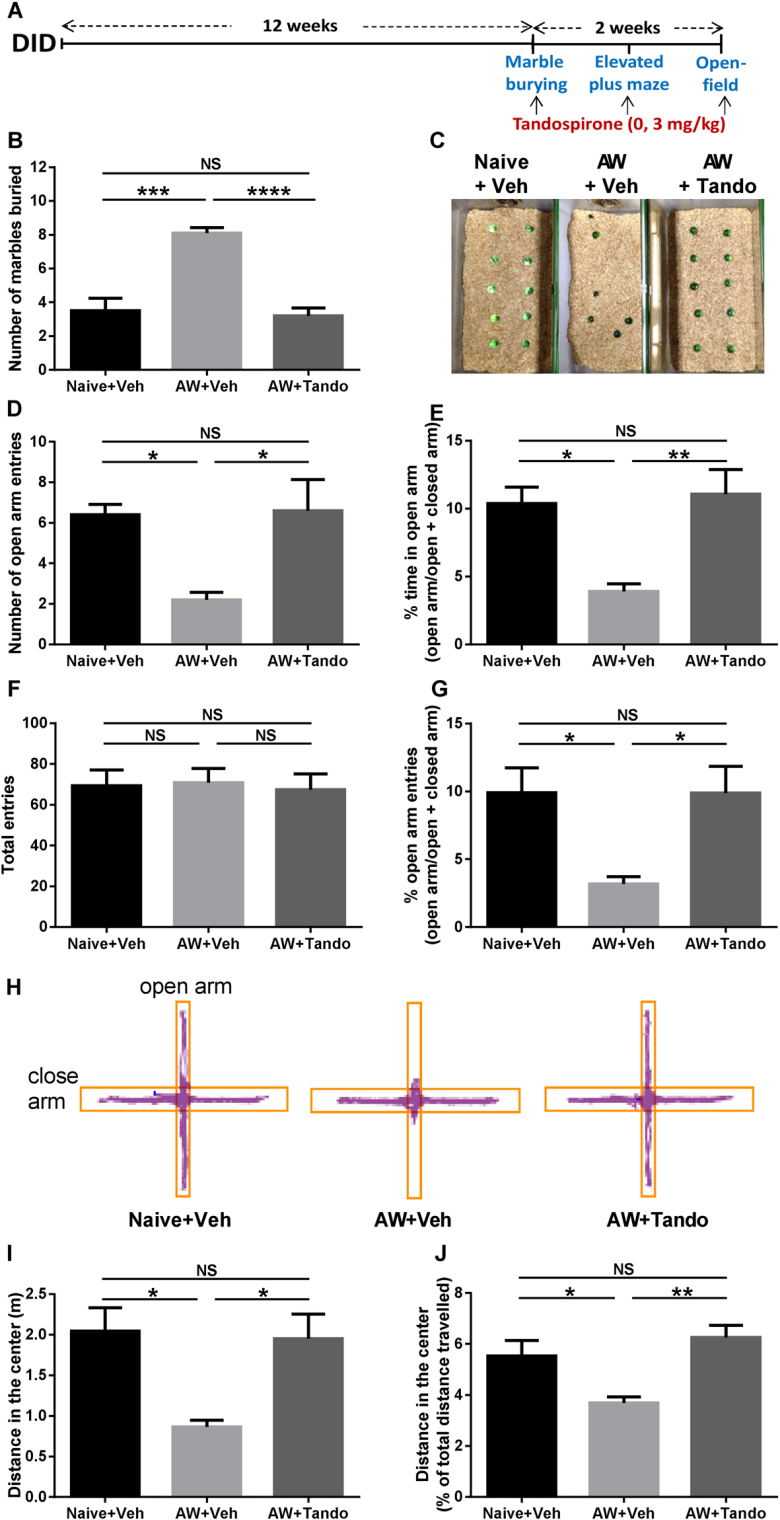
Tandospirone prevents withdrawal-induced anxiety following long-term chronic binge-like alcohol consumption. (**A**) Experimental design of anxiety-like behavioral testing in the marble burying (**B**–**C**), elevated-plus-maze (**D**–**H**) and the open-field (**I**–**J**) following 12 weeks of binge-like alcohol consumption in the drinking-in-the-dark (DID). Alcohol-naive animals were treated with vehicle (Naïve + veh) and alcohol-withdrawn animals with either vehicle (AW + Veh) or Tandospirone (AW + Tando). (**B**) Marble burying test showing an increased number of marble buried by alcohol-withdrawn mice that is prevented by tandospirone pretreatment. (**C**) Representative photograph of marble burying behavior for the different groups. (**D**–**E**) Alcohol-withdrawal reduces the number of entries (**D**) and the percentage of time spent (open arm/ open arm + closed arm, **E**) in the open arm of the elevated plus maze in vehicle- but not tandospirone-pretreated mice, as compared to naive mice treated with vehicle. (**F**) The total number of entries in the different arms of the elevated-plus-maze is not altered by either alcohol-withdrawal or tandospirone pretreatment. (**G**) The number of open-arm entries expressed as a percentage of total entries (open arm/open arm + closed arm) is reduced in alcohol-withdrawn mice, which is prevented by tandospirone pretreatment, as compared to naive mice treated with vehicle. (**H**) Representative picture of the exploratory behaviour for the different groups. (**I**–**J**) Alcohol-withdrawal reduces the distance travelled in the center of the open-field (**I**) and the distance in the center as a percentage of the total distance in the open-field (**J**) in vehicle- but not tandospirone-pretreated mice, as compared to naive mice treated with vehicle. Data are presented as mean ±S.E.M; n = 5 mice/group. *p < 0.05; **p < 0.01; ***p < 0.001; ****p < 0.0001; NS: non-significant (One-way ANOVA followed by Bonferroni *post-hoc* analysis).

To evaluate the contribution of 5-HT_1A_ receptors in anxiety-like behavior following withdrawal from long-term binge-like drinking, we investigated the effects of the 5-HT_1A_ partial agonist tandospirone (3 mg/kg) on mice experiencing a 24-h withdrawal from alcohol in a battery of behavioral tests (marble burying, elevated-plus-maze and open-field) (Fig. 1A). In the marble burying test, we observed a significant increase in anxiety-related marble digging behavior in alcohol-withdrawn mice compared to naive mice, and this increase was prevented by tandospirone pre-treatment (Fig. 1B). A representative image of the marble buried for the three groups is shown (Fig. 1C). In the elevated-plus-maze test, the number of entries and the percent of time spent in the open-arm were significantly reduced in ethanol-withdrawn animals compared to naive animals, and this anxiety-related avoidance of heights/open space was abolished by tandospirone pre-treatment (Fig. 1D,E). Since the total number of entries was not different between naive, alcohol withdrawn and tandospirone-treated alcohol withdrawn mice (Fig. 1F), the open arm entries expressed as the percentage of the total entries was also significantly reduced in vehicle-treated alcohol-withdrawn mice compared to naive mice, with tandospirone treatment also preventing this reduction (Fig. 1G). It demonstrates that the anti-anxiety effect of tandospirone following alcohol withdrawal is not mediated by alterations in locomotor activity. A representative image of the ambulatory behavior in the elevated-plus-maze is shown for the three groups (Fig. 1H). Similarly, in the open-field test, the distance travelled in the center was reduced in alcohol-withdrawn mice compared to naive animals, and this reduction was absent in tandospirone treated animals (Fig. 1I). The total distance travelled was not significantly different between groups (Figure S1a) and therefore, the distance travelled in the center expressed in percentage of the total distance travelled was significantly reduced in vehicle-treated alcohol withdrawn mice but not in tandospirone-treated alcohol withdrawn mice (Fig. 1J). Again, it reveals that the anxiolytic effect of tandospirone is not mediated by alterations in locomotor activity. Taken together, these results demonstrate that the anxiogenic effects of alcohol-withdrawal after 12 weeks of binge-like consumption in the DID are totally reversed by the selective partial activation of 5-HT_1A_ receptors by tandospirone.

### Partial activation of 5-HT_1A_ receptors by tandospirone reduces binge-like alcohol consumption in long-term ethanol consuming mice in the DID

**Figure 2:**
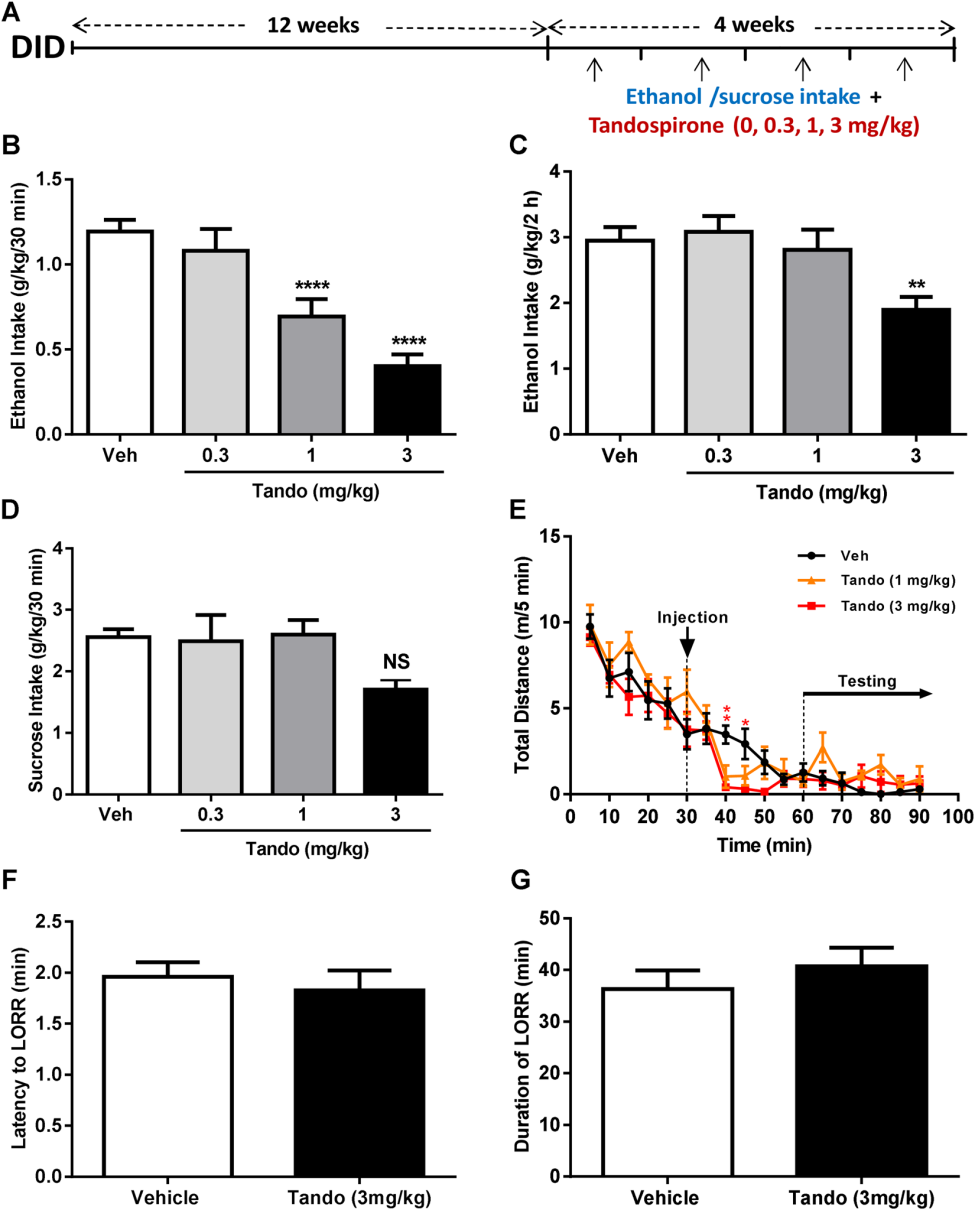
Tandospirone reduces the maintenance of long-term binge-like alcohol consumption. (**A**) Experimental design of the testing of tandospirone on alcohol intake following 12 week of alcohol binge-like consumption in DID. (**B**,**C**) Tandospirone pre-treatment (Veh, 0.3, 1 and 3 mg/kg) reduces 30-min (**B**) and 2-h (**C**) ethanol intake (n = 11 mice). (**D**) Tandospirone pre-treatment (Veh, 0.3, 1 and 3 mg/kg) has no effect on 30-min sucrose intake (n = 10 mice). Data are presented as mean intake (g/kg) ±S.E.M. **p < 0.01; ****p < 0.0001; NS: non-significant (One-way ANOVA with repeated measures followed by Bonferroni *post-hoc* analysis). (**E**) Tandospirone reduces the locomotor activity immediately after injection but has no effect on locomotion during the behavioural or intake testing period. Data are presented as mean distance travelled per 5 min ± S.E.M; n = 6 mice/group. *p < 0.05; **p < 0.01 for the 3 mg/kg dose compared to vehicle (Two-way ANOVA followed by Bonferroni *post-hoc* analysis). (**F–G**) Tandospirone has no effect on the latency to (**F**) or the duration of (**G**) the Loss-Of-Righting-Reflex (LORR) induced by a sedative dose of ethanol. Data are presented as mean ±S.E.M; n = 6 mice/group (Unpaired two-tailed Student *t*-test).

We assessed whether the anti-anxiety effect of partial activation of 5-HT_1A_ receptors during daily withdrawal in the DID could affect the levels of alcohol intake. We tested the effect of tandospirone pre-treatment on the levels of binge-like alcohol intake (Fig. 2A). Compared to vehicle, tandospirone significantly reduced alcohol intake at 30-min (Fig. 2B), and 2 h (Fig. 2C). To evaluate the contribution of any non-specific effects in the efficacy of tandospirone to reduce alcohol consumption, we further tested the effect of tandospirone on sucrose intake, general locomotor activity and alcohol metabolism. We did not observe any alterations to the consumption of sucrose at 30 min (or 2 h, Figure S1b) with any dose of tandospirone administered (0, 0.3, 1 and 3 mg/kg) (Fig. 2D). Additionally, while a slight reduction in locomotor activity was observed at 10 and 15 minutes after tandospirone injection, ambulatory behavior was not significantly altered by tandospirone after 30 min post-injection, *i.e*. during the period corresponding to the testing of alcohol/sucrose intake or anxiety (Fig. 2E). Finally, assessing tandospirone non-specificity on alcohol metabolism revealed no effect on the latency (Fig. 2F) and duration (Fig. 2G) of the loss-of-righting-reflex, as compared to vehicle. Together, these results demonstrate that tandospirone specifically reduces the maintenance of binge-like alcohol drinking behaviors following long-term exposure in the DID.

### Chronic activation of 5-HT_1A_ receptors reverses the neurogenic deficits induced by long-term binge-like alcohol consumption in the DID

**Figure 3:**
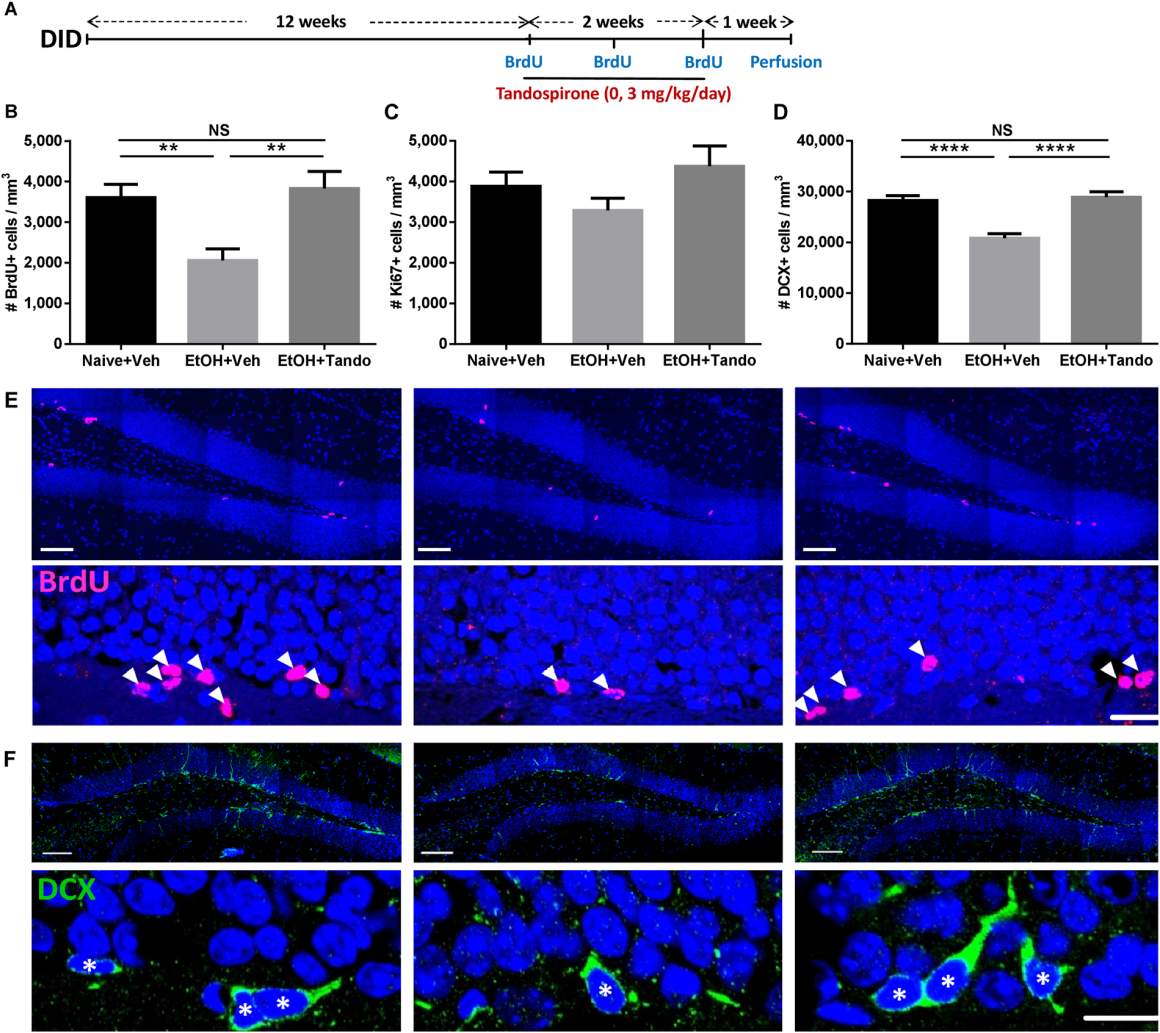
Tandospirone reverses the alcohol-induced deficits in hippocampal neurogenesis. (**A**) Experimental design of chronic tandospirone and BrdU injections. After 12 weeks of DID, alcohol-exposed (EtOH) and naive (Naive) mice received a first BrdU injection (150 mg/kg, i.p.). Naive mice were then chronically treated with vehicle (Naive + Veh) and alcohol-exposed mice were chronically treated with either vehicle (EtOH + Veh) or Tandospirone (3 mg/kg/day) (EtOH + Tando), for 2 weeks. Mice were injected with BrdU (150 mg/kg, i.p.) at the end of each treatment week. Experiment ended after an extra week of DID and neurogenesis was assessed in n = 5 animals/group; 6 dentate gyri/animal. (**B**) Long-term chronic alcohol binge-like drinking in the DID reduces the density of BrdU-immunoreactive (BrdU+) cells in the dentate gyrus of the dorsal hippocampus and this effect was totally prevented by a two-week chronic treatment with tandospirone, as compared to ethanol naive mice treated with vehicle. (**C**) The density of Ki67-immunoreactive cells (Ki67+) is not affected by alcohol consumption or tandospirone treatment. (**D**) Long-term chronic alcohol binge-like drinking decreases the density of DCX-immunoreactive (DCX+) immature neurons in the dentate gyrus, and this reduction was prevented by a chronic tandospirone treatment, as compared to ethanol naive mice treated with vehicle. Data are presented as mean density of cells per mm^3^ of granular layer ±S.E.M; n = 6 mice/group, 5 dentate gyri/mouse. **p < 0.01; ****p < 0.0001, NS: non-significant (One-way ANOVA followed by Bonferroni *post-hoc* analysis). (**E,F**) Left, middle and right vertical panels correspond to Naive + Veh, EtOH + Veh and EtOH + Tando, respectively. (**E**) Representative micrographs of the effect of long-term ethanol consumption and chronic tandospirone treatment on the density of BrdU+ cells in the sub-granular/granular layer of the dentate gyrus. Top horizontal panel shows lower magnification images, scale bar = 50 μm. Bottom horizontal panel shows higher magnification images, scale bar = 20 μm. Blue: DAPI, purple: BrdU, white arrowhead: BrdU+ cells. (**F**) Representative micrographs of the effect of long-term ethanol consumption and/chronic tandospirone treatment on the density of DCX+ immature neurons in the sub-granular/granular layer of the dentate gyrus. Top horizontal panel shows lower magnification images, scale bar = 100 μm. Bottom horizontal panel shows higher magnification images, scale bar = 10 μm. Blue: DAPI, green: DCX, white asterisk: DCX+ immature neurons.

Since chronic treatment with tandospirone was previously shown to increase hippocampal neurogenesis and prevent stress-induced deficits in neurogenesis in rats^34,35^, we investigated the effect of a 2-week chronic treatment (3 mg/kg/day) on the incorporation of BrdU, the expression of the proliferation marker, Ki67 and the marker of immature neurons, doublecortin (DCX) in the dentate gyrus of mice long-term exposed to alcohol-binge-like consumption in the DID (Fig. 3A**)**. We observed that prolonged binge-like alcohol consumption significantly reduced the density of BrdU immunoreactive (BrdU+) cells in the dentate gyrus of alcohol-withdrawn mice compared to naive mice, and this reduction was prevented by chronic tandospirone treatment (Fig. 3B**)**. Quantification of Ki67 immunoreactive (Ki67+) cells revealed no effect of alcohol exposure or tandospirone treatment on the density of proliferating cells compared to vehicle-treated naïve animals (Fig. 3C). However, long-term alcohol exposure significantly reduced the density of DCX immunoreactive (DCX+) immature neurons compared to naive animals, and chronic tandospirone treatment significantly reverses this reduction (Fig. 3D). Representative micrographs of the effect of prolonged alcohol binge-like consumption and/or the effect of chronic tandospirone treatment on the density of BrdU and DCX immunoreactive cells in the dentate gyrus are shown (Fig. 3E, F, respectively).

**Figure 4:**
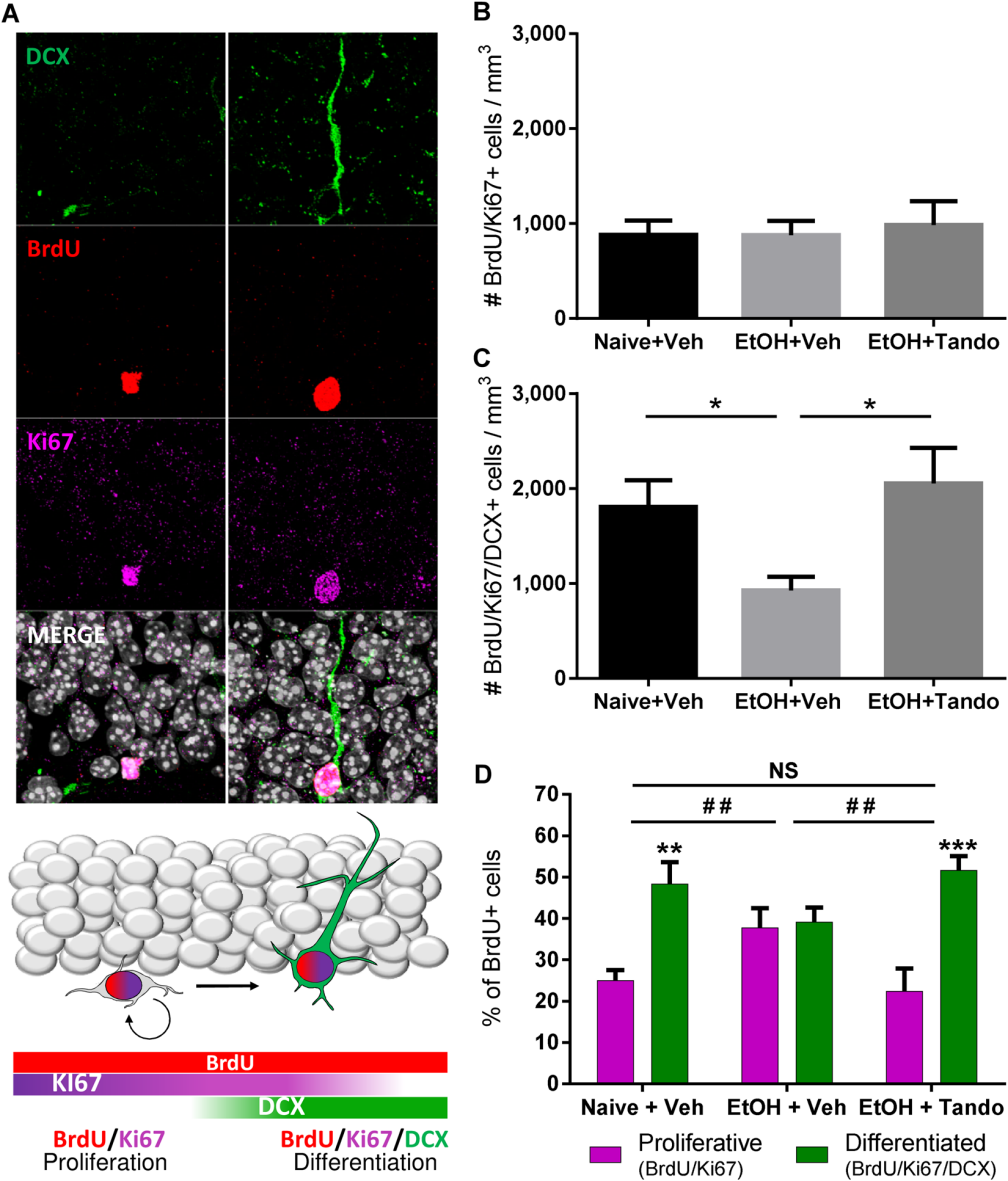
Tandospirone rescues alcohol-induced alterations in neuronal differentiation in the hippocampus. (**A**) Proliferation and neuronal fate specification/differentiation of BrdU+ cells (red) coexpressing DCX (green) and Ki67 (purple) and counterstained with DAPI (white). The left panel shows a BrdU+ cell only co-expressing Ki67 and the right panel a BrdU+ cell co-expressing both Ki67 and DCX. The bottom drawing illustrates the neuronal fate specification from BrdU/Ki67+ proliferating (non-differentiated) progenitors to BrdU/Ki67/DCX+ differentiated neuroblasts. (**B**) The density of BrdU/Ki67+ proliferating progenitors is not affected by alcohol consumption or tandospirone treatment. (**C**) Long-term chronic alcohol binge-like drinking decreases the density of BrdU/Ki67/DCX+ differentiated neuroblasts in the dentate gyrus, and this effect was totally abolished by chronic tandospirone treatment. Data are presented as mean density of cells per mm^3^ of granular layer ±S.E.M; n = 6 mice/group, 5 dentate gyri/mouse. *p < 0.05 (One-way ANOVA followed by Bonferroni *post-hoc* analysis). (**D**) Long-term alcohol binge-like consumption increases the proportion of BrdU progenitors that are proliferating, but reduces the neuronal differentiation of these progenitors. A chronic treatment with tandospirone totally restores a normal neuronal differentiation process in long-term ethanol-exposed mice, compared to ethanol naive mice treated with vehicle. Data are presented as mean percentage of BrdU+ cells ± S.E.M. **p < 0.01; ***p < 0.001, compared to proliferative progenitors (Two-way ANOVA with Bonferroni *post-hoc* test); ^##^p < 0.01 (χ^2^ analysis of the distribution of the BrdU+ cells within proliferation and differentiation stages).

To further assess whether the neuronal differentiation process was impaired by alcohol or tandospirone during the last 2 weeks of DID, we investigated the distribution of proliferation and immature neuron markers within the BrdU^+^ cell population. We quantified the density of BrdU^+^ cells co-expressing Ki67 (BrdU/Ki67^+^: proliferating progenitors) or both Ki67 and DCX (BrdU/Ki67/DCX^+^: differentiated neuroblast) (Fig. 4A). Quantification of the density of BrdU/Ki67^+^ cells revealed no effect of alcohol exposure or tandospirone treatment compared to naive animals (Fig. 4B). However, alcohol exposure significantly reduces the density of BrdU/Ki67/DCX^+^ cells compared to naive animals. Chronic treatment with tandospirone reversed this reduction (Fig. 4C). Furthermore, analysis of the distribution of the different cell population markers within BrdU^+^ cells showed that long-term alcohol exposure alters the neuronal fate specification compared to naive animals, and chronic tandospirone treatment in alcohol-exposed animals restored a normal neurogenesis process (Fig. 4D). These results demonstrate that chronic partial activation of 5-HT_1A_ receptors by 2-week tandospirone treatment is sufficient to totally reverse the neurogenic and neuronal differentiation impairments elicited by 15-weeks of alcohol binge-like consumption in the DID.

## Discussion

The present findings identify the importance of 5-HT_1A_ receptors in alcohol withdrawal-induced anxiety, anxiety-driven alcohol drinking and alcohol-induced neurogenic deficits following long-term chronic binge-like voluntary consumption in the DID paradigm. Previous studies using forced alcohol administration, including liquid diet, gavage or vapors, have suggested the involvement of 5-HT_1A_ in anxiety^36–38^ and stress-induced anxiety^39^ following acute or protracted withdrawal from chronic alcohol exposure. However, these studies have only suggested the contribution of 5-HT_1A_ receptors using the partial-agonist buspirone, a non-selective drug that has been later shown to possess an antagonist activity at dopamine D2 receptors^40^, which could explain the controversial results showing sedative, anxiolytic, anxiogenic or ineffective properties of buspirone in various behavioral paradigms^26,41^. Similarly, clinical testing of buspirone for the treatment of alcohol abuse or the management of withdrawal-induced anxiety has led to conflicting results^42,43^, therefore raising uncertainty towards the efficacy of partial activation of 5-HT_1A_ receptors in the treatment of alcohol use disorders.

The anxiolytic drug tandospirone is a 5-HT_1A_ receptor partial agonist (60% efficacy) having two to three orders of magnitude less potency for adrenergic, muscarinic, dopamine or any other 5-HT receptors^32^. Tandospirone’s binding sites are predominantly located in the hippocampus (dentate gyrus), lateral septum, entorhinal cortex, interpeduncular nucleus and dorsal raphé (DR) nucleus^44^. The anxiolytic action of tandospirone in rodents has been observed in conflict^45^, fear-conditioned freezing^46^ and marble burying tests^47^, yet a recent report failed to observe an anxiolytic action of tandospirone (3, 10 and 30 mg/kg) on rat basal anxiety in the elevated-plus-maze^48^. Here, we have established the efficacy of tandospirone (3 mg/kg) to alleviate alcohol withdrawal-induced anxiety not only in marble burying and open-field tests, but also in the elevated-plus-maze. Whether the difference between the aforementioned study and our work is related to the administration route (oral *vs* intraperitoneal in our study) or reveals a specific effect of tandospirone on alcohol withdrawal-induced-anxiety following long-term DID still needs to be determined.

Chronic alcohol exposure increases the net excitation of dorsal raphe neurons^38^ and upregulates 5-HT_1A_ autoreceptor expression^16^ and function^15^, which has been postulated to contribute to anxiety during alcohol-withdrawal^38^. Conversely, tandospirone decreases the firing rate of DR 5-HT neurons and desensitizes 5-HT_1A_ autoreceptors without affecting hippocampal 5-HT_1A_ heteroreceptors^49^. Hence, the observed effects of tandospirone may be mediated by its action on 5-HT_1A_ autoreceptors, normalizing 5-HT neurotransmission in the DR and reducing both withdrawal-induced anxiety and the maintenance of alcohol binge-like drinking behavior in mice consuming alcohol from early-adolescence through late adulthood. However, the anti-conflict effect of tandospirone is likely mediated by 5-HT_1A_ receptors located in the dorsal hippocampus^45^. Although 5-HT_1A_ autoreceptors have been demonstrated to be developmentally necessary for the establishment of normal anxiety-like behaviour^12^, there is compelling evidence to suggest that 5-HT_1A_ receptor agonists exert their anxiolytic effects postsynaptically in the hippocampus^14,50–52^. Therefore, further work using local injections in the dorsal raphe or the hippocampus is needed to determine whether the anxiolytic effect of tandospirone in alcohol-withdrawn mice is mediated by 5-HT_1A_ auto- or heteroreceptors. Alternatively, the development of biased 5-HT_1A_ receptor partial agonists displaying high selectivity for either auto or heteroreceptors represents a potential strategy to resolve this question^53^.

Raphe-hippocampal circuitry is complex, with the dorsal and ventral hippocampus differentially innervated by the dorsal as well as median raphe, with collateral interconnections within the ventral/dorsal hippocampus and the dorsal/median raphe^54^. In addition, the neuronal activity of these regions is governed by cortico-striatal loops, forming a complex neural network that encodes the different aspect of reward processing^55^. As a result, the role of the hippocampus in reward processing appears to be topographically organized, with the dorsal hippocampus likely playing a more prominent role in contextual/spatial memory^56^ and the ventral hippocampus more involved in emotional/motivation-related memory^57^. Thus, tandospirone might act presynaptically on 5-HT_1A_ autoreceptors as a full agonist in the dorsal^58^ and as a partial agonist in the median raphe^59^ to differentially inhibit 5-HT release in target brain regions, including the ventral/dorsal hippocampus, as well as the prefrontal cortex, the amygdala or the bed nucleus of the stria terminalis, brain regions in which serotonin signaling has been widely implicated in both alcohol drinking behaviors and withdrawal-associated emotional deficits^60^. Tandospirone could also act postsynaptically as a full agonist on 5-HT_1A_ heteroreceptors located in different neuronal subtypes (glutamate versus GABA neurons) in those brain regions to modulate their excitability/activity^58^. Further work using recent advances in optogenetics and/or chemogenetics would help to dissect the region- and circuit-specific contribution of 5-HT_1A_ auto- and heteroreceptors in alcohol drinking behavior and associated emotional or neurogenic deficits.

Over previous decades, a large body of preclinical and clinical evidence has linked 5-HT homeostasis deficits to hippocampal neurogenesis impairments and depression-related disorders. More recently, adult hippocampal neurogenesis has also been reported to modulate anxiety-like behavior in rodents^14,61^. Repeated cycles of binge-like alcohol exposure for relatively short periods of time (4 days to 7 weeks), using forced-consumption (intra-gastric gavage or ethanol vapors), alcohol-containing diet or sweetened-solution fading procedures, produces anxiety-like behavior concomitant to reduced progenitor proliferation (BrdU incorporation and Ki67 labeling) and impaired neuronal differentiation (DCX labeling) in the dentate gyrus^62^. Similarly, 12 days of voluntary binge-like consumption of sweetened ethanol was shown to produce anxiety- and depression-like behavior, and reduce the density of BrdU+ proliferating cells and DCX+ immature neurons^63^. Here, we showed that long-term voluntary binge-like consumption of ethanol in the DID, from early-adolescence through late adulthood, not only leads to significant reductions in progenitor proliferation in the DG but, once they have proliferated, their neuronal fate specification is altered. Interestingly, we were able to reverse both proliferation and differentiation deficits with a chronic tandospirone treatment for the last two weeks of a 15-week exposure in the DID. It suggests that impairments in neurogenesis following long-term binge-like voluntary consumption of alcohol might be mediated, at least in part, by a 5-HT_1A_ receptor-dependent mechanism. One explanation could be that chronic alcohol exposure might reduce the expression and/or function of 5-HT_1A_ heteroreceptors within the dentate gyrus, as previously described^16^. This reduction in 5-HT_1A_ receptors expression or function could result from a reduction in BDNF/TrkB signaling^64^ induced by chronic alcohol exposure^63^. In turn, decreased 5-HT_1A_ function might alter CREB phosphorylation pathway activation, which could lead to decreased neurogenesis, and increased anxiety-like behaviour^14^. On the other hand, an upregulation of 5-HT_1A_ autoreceptors might contribute to the effect seen on alcohol drinking and anxiety related behavior. Hence, tandospirone might act as a partial agonist on 5-HT_1A_ autoreceptors and a full agonist on 5-HT_1A_ heteroreceptors, to compensate for altered 5-HT_1A_ receptor signaling in the dorsal raphe and the hippocampus, reduce anxiety-like behavior and correct the neurogenic deficits, respectively. Since similar alterations in hippocampal neurogenesis have been observed following chronic binge-like alcohol administration by gastric gavage in rats, with these changes associated with concomitant cognitive impairments^65^, further work is required to determine whether neurogenic deficits following long-term DID lead to cognitive deficits and whether these deficits are mediated by a 5-HT_1A_ receptor-dependent mechanism.

## Methods

### Animals and housing

Five-week-old male C57BL/6 J mice (ARC, WA, Australia) were individually housed under reverse light cycle conditions (lights off at 9:00 am) in a climate-controlled room with *ad libitum* access to food and water. Following 1 week of habituation to the housing conditions, mice were offered alcohol during the drinking sessions. All experimental procedures were approved by The University of Queensland and The Queensland University of Technology Animal Ethics Committees and complied with the policies and regulations regarding animal experimentation and other ethical matters, in accordance with the Queensland Government Animal Research Act 2001, associated Animal Care and Protection Regulations (2002 and 2008), as well as the Australian Code for the Care and Use of Animals for Scientific Purposes, 8th Edition (National Health and Medical Research Council, 2013).

### Drugs and Chemicals

Tandospirone (Sigma-Aldrich, NSW, Australia) was dissolved in 2% dimethyl sulfoxide (DMSO) and saline (0.9% NaCl). The 20% alcohol (v/v) solution was prepared using 100% food grade ethyl alcohol (Recochem, SA, Australia) and filtered water. The 5% sucrose (w/v) (Chem supply, SA, Australia) solution was prepared in filtered water. BrdU (5-BromoUracil deoxyriboside, Sigma-Aldrich) was dissolved in 1% DMSO and 0.1 M phosphate buffered saline (PBS, pH 7.4).

### Drinking-In-the-Dark (DID) paradigm

We adapted the Drinking-In-the-Dark (DID) model of binge-like alcohol^8^ or sucrose consumption with long-term exposure as previously described^29,66^. Mice were given access to one bottle of 20% (v/v) alcohol for a 2 h period (12 pm to 2 pm), 3 h into the dark cycle, Monday to Friday. Filtered water was available at all other times. The alcohol solution was presented in 50 ml plastic falcon tubes (Corning Centristar, NY, USA) fitted with rubber stoppers and a 6.35 cm stainless-steel sipper tube with double ball bearings. Alcohol containing tubes were weighed prior to and 30 min and 2 h after presentation. Mouse weights were measured daily to calculate the adjusted g/kg intake. We also measured binge-like long-term sucrose consumption by adapting the DID paradigm: mice were given access to one bottle of 5% (w/v) sucrose instead of alcohol.

### Acute tandospirone testing

Following 12 weeks of DID, mice consuming stable baseline levels of 20% alcohol (Figure S1c) were divided in two groups for the testing of acute tandospirone on (1) anxiety or (2) the maintenance of alcohol and sucrose drinking, 24 h after the last drinking session.

- For the anxiety-related behavioral experiments, tandospirone (vehicle or 3 mg/kg) was intraperitoneally (i.p.) administered 30 min prior to behavioral testing. Each of the 3 tests was conducted 1 week apart, over 3 weeks. In all the tests, alcohol-withdrawn mice were assigned to one of two treatment groups: vehicle (AW + Veh) or tandospirone 3 mg/kg (AW + Tando) and the alcohol naive age-matched mice were assigned to the vehicle group (Naive + Veh), n = 5–6 animals/group.
- For the experiments on the maintenance of alcohol or sucrose drinking, tandospirone (vehicle, 0.3, 1, 3 mg/kg) was intraperitoneally (i.p.) administered to mice consuming stable levels of 20% ethanol (n = 11) or 5% sucrose (n = 10). The drug testing was carried out seven days apart, using a Latin square design, thus each animal randomly received one of the 4 doses each week. After 4 weeks, each animal has received all the 4 doses and serves as its own control. Tandospirone was administered 30 min before the presentation of the bottles.

### Anxiety-related behavior

For the testing of withdrawal-induced anxiety-like behavior, experiments were carried out 24 h after the last drinking session of the week. *Marble burying* was performed in novel individual plastic cages (21 × 38 × 14 cm) containing 5-cm thick sawdust bedding. Ten glass marbles (diameter 10–12 mm) were arranged on the bedding evenly spaced in 2 rows of 5 marbles. After 20 min, the number of unburied marbles was averaged from counting by two experimenters blind to the treatments. A marble covered at least two-third (2/3) of its size by saw dust was considered as “buried”. *Elevated-plus-maze* was carried out in an apparatus consisting of four arms (35 cm × 5 cm), elevated 50 cm above the floor. The closed arms were enclosed with 40 cm high walls. The experiment was conducted for 5 min, with initial mouse placement in the center, facing the open arm^67^. The number of entries and time spent in each arm was recorded using ANY-maze tracking software (Stoelting, IL, USA). *Open-field* was performed in an open arena of 30 × 30 cm. The floor was divided into 16 equal squares (7 × 7 cm) and a central region of 10 × 10 cm was considered as the center. Mice were initially placed in one corner, and allowed to explore freely for 10 min. The number of entries and the time spent in the center were recorded using the ANY-maze software.

### General locomotor activity

Locomotor activity was performed as previously described^29,66^. The study was run in 4 daily 2 h sessions. After habituation (day 1–3), testing was conducted on day 4. Alcohol naive mice (13–15 week-old) were assigned to one of three treatment groups: vehicle or tandospirone (1 or 3 mg/kg), n = 6 animals per group. After 60 min, animals were i.p. injected with the assigned treatment and the locomotor activity was recorded for 60 min using ANY-Maze tracking software. Data was collected across the entire 2 h session and recorded as distance travelled in meters per 5 min.

### Loss of Righting Reflex (LORR)

LORR experiment was performed as previously described^29,66^. A sedative dose of 3.2 g/kg (20% v/v, i.p.) of ethanol was administered to test the effects of tandospirone on alcohol metabolism. Ethanol naive mice were divided into two groups that either received saline or tandospirone (3 mg/kg, i.p.), 30 min before ethanol was administered. The latency to LORR was recorded as the time from ethanol injection to the time the mouse was unable to right itself 3 times within a 15-second period from the supine position. Duration of LORR was recorded as the time elapsed until it recovered and was able to right itself 3 times within a 15-second period.

### Chronic tandospirone testing on neurogenesis

Following 12 weeks of alcohol drinking in the DID, Tandospirone 3 mg/kg/day was i.p. injected immediately after each drinking session to prevent any effect on alcohol consumption levels (Figure S1d) for 2 weeks. A total of three injections of BrdU (150 mg/kg, i.p.) were performed over the 2 week treatment (days 0, 7 and 15). This non-toxic dose has been reported to label all actively dividing precursors in the mouse subgranular zone^68^. Twenty-four hours after the last BrdU injection, animals were transcardially perfused with 4% paraformaldehyde. Brains were harvested and postfixed overnight at 4 °C, incubated in sucrose 20–30%, embedded in Optimal Cutting Temperature (OCT, Tissue-Tek) medium, and kept at −80 °C until histology and immunohistochemistry processing. Twenty micron-thick coronal cryostat sections were collected and kept floating in ice-cold PBS. After 3 thorough washes in PBS, slices were immersed in ice-cold 10% methanol-PBS solution for 5 min. After 3 washes in citrate buffer solution (10 mM citrate buffer, 0.05% Tween 20, pH 6.0) at room temperature, sections were incubated in a 37 °C pre-heated citrate buffer solution and placed at 95 °C for 20 min, rinsed 3 times in PBS and incubated overnight in blocking solution (4% normal goat serum-NGS, 1% bovine serum albumin- BSA, 0.3% Triton X100 and 0.05% Tween 20). Sections containing the dorsal hippocampus (Bregma −1.5 mm to −2 mm) were incubated overnight at 4 °C with primary antibodies: rabbit anti-DCX (Abcam #18723, 1:500); mouse anti-Ki67 (BD bioscience #550609, 1:20) and rat anti-BrdU (Abcam #6326, 1:200) and with corresponding secondary antibodies, for 2 h at room temperature: goat anti-rabbit-Alexa488; goat anti-mouse-CY5; goat anti-rat-Biotin (Thermofisher Scientific, 1:500) and 30 min at room temperature with Streptavidin-Cy3 (Thermofisher Scientific, 1:1000). Sections were mounted in Prolong gold antifade mountant with DAPI (Thermofisher Scientific).

### Imaging and Analysis

Whole dentate gyri of 3 coronal sections per animals (n = 5–6 animals/group) were imaged on a Nikon/Spectral Spinning Disk confocal microscope in mosaics using a 40× oil-immersion objective (NA 1.35), with a z-step of 0.5 μm. Four-channel mosaic images (.nd2) were deconvolved using Huygens professional v16.10 (Scientific Volume Imaging, The Netherlands) with iteration number set at 100, quality threshold at 0.001, signal to noise ratio at 15 for the 4 channels and converted in .tif for subsequent quantification in Neurolucida 360 (MBF Bioscience). Counting of BrdU+, DCX+, Ki67+, BrdU/Ki67+ and BrdU/Ki67/DCX+ cells was performed by an experimenter blind to the treatment, averaged per animal and plot as mean ± SEM for each group. Density of counted cells was normalized to the volume of granular cell layer sampled in each group. Representative images were taken on a Olympus FV1200 confocal microscope using a 60× oil-objective (NA 1.35), x3.0 numerical zoom and 0.5 z-step.

### Statistics

GraphPad Prism 7 (Graph Pad Software Co., CA, USA) was used for all statistical analysis. Comparisons between groups were statistically analysed using *t*-test, one-way or two-way ANOVA followed by a Bonferroni multiple comparisons *post-hoc* test. A p value < 0.05 was considered significant. All values are expressed as the mean ± SEM. Distribution of the different markers within BrdU-immunoreactive cell population was compared between groups using the Chi-squared (χ^2^) test. A p value < 0.05 was considered significant. Detailed statistical analysis is provided in Table S1.

## Electronic supplementary material

Supplementary material
