# Supplementary material for: 5-HT1A receptor-dependent modulation of emotional and neurogenic deficits elicited by prolonged consumption of alcohol
Source: Sci Rep. 2018 Feb 1;8:2099. doi: 10.1038/s41598-018-20504-z (PMC5794771; doi:10.1038/s41598-018-20504-z)
Supplement: Supplementary file 1 — Supplementary material [file 41598_2018_20504_MOESM1_ESM.doc]

Title: 5-HT_1A_ receptor-dependent modulation of emotional and neurogenic deficits elicited by prolonged consumption of alcohol.

**Arnauld Belmer^1, 2#^, Omkar L. Patkar^1, 2#^, Vanessa Lanoue^3^ and Selena E. Bartlett^1, 2*^**

^1^Translational Research Institute, Queensland University of Technology, Brisbane, 4100, Australia

^2^Institute of Health and Biomedical Innovation (IHBI), Queensland University of Technology, 4100, Brisbane, Australia

^3^Queensland Brain Institute, University of Queensland, Brisbane, 4100 Australia.

^*^Selena.bartlett@qut.edu.au

**^#^**These authors contributed equally to this work

**Supplementary information**


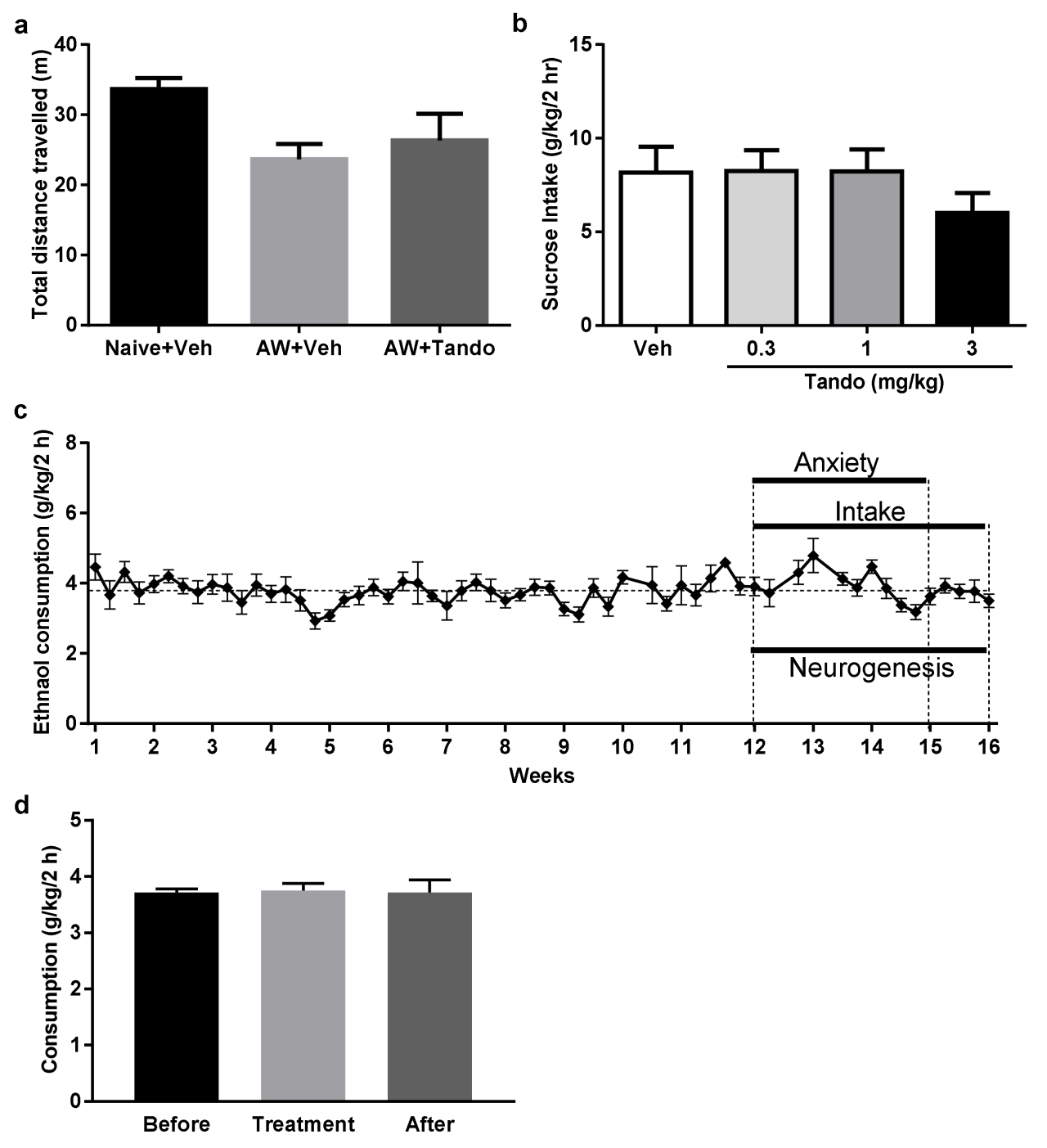
**Fig. S1**

**Figure S1: Tandospirone has no non-specific effects on locomotor activity or sucrose consumption in mice consuming stable levels of ethanol. (a)** Ethanol withdrawal and tandospirone treatment do not affect the total distance travelled in the open-field. Data are presented as mean total distance travelled in meters ± S.E.M. One-way ANOVA analysis revealed no significant effect of alcohol withdrawal or tandospirone treatment (F (2, 12) = 3.781; p=0.053). **(b)** Tandospirone has no effect on 2h sucrose intake in long-term sucrose consuming mice in the DID (Data are presented as mean sucrose intake in g/kg ± S.E.M. One-way ANOVA analysis revealed no significant effect of tandospirone on 2h sucrose intake (F (3, 27) = 0.7388; p=0.5381). **(c)** The effect of tandospirone on anxiety, ethanol intake and neurogenesis was assessed in mice consuming stable baseline levels of ethanol over 12 to 16 weeks of DID. Average mean ethanol intake was 3.8 ± 0.4 g/kg/2h. **(e)** Chronic tandospirone (3 mg/kg/day) treatment immediately after each daily drinking session had no effect on ethanol intake levels. Data are presented as mean consumption in g/kg/2h. ± S.E.M. One-way ANOVA, F (2, 62) = 0.04377; p=0.9572

.

Table S1: Statistical analysis

| Fig. | Test | F value | | P value | Significance | Post-hoc | Multiple comparison | n | P value | Significance |
| --- | --- | --- | --- | --- | --- | --- | --- | --- | --- | --- |
| 1B | One-way ANOVA | F(2,12) = 25.86 | | <0.0001 | ******** | Bonferroni | Naive+Veh vs. AW+Veh | 5 | 0.0002 | ******* |
|  |  |  |  |  |  |  | Naive+Veh vs. AW+Tando |  | >0.9999 | **ns** |
|  |  |  |  |  |  |  | AW+Veh vs. AW+Tando |  | <0.0001 | ******** |
| 1D |  | F (2, 12) = 6.71 | | 0.0111 | ***** |  | AW+Veh vs. AW+Tando | 5 | 0.0211 | ***** |
|  |  |  |  |  |  |  | AW+Veh vs. Naive+Veh |  | 0.0278 | ***** |
|  |  |  |  |  |  |  | AW+Tando vs. Naive+Veh |  | >0.9999 | **ns** |
| 1E |  | F (2, 12) = 9.106 | | 0.0039 | ****** |  | AW+Veh vs. AW+Tando | 5 | 0.0067 | ****** |
|  |  |  |  |  |  |  | AW+Veh vs. Naive+Veh |  | 0.0133 | ***** |
|  |  |  |  |  |  |  | AW+Tando vs. Naive+Veh |  | >0.9999 | **ns** |
| 1F |  | F (2, 12) = 0.05169 | | 0.9498 | **ns** |  | AW+Veh vs. AW+Tando | 5 | >0.9999 | **ns** |
|  |  |  |  |  |  |  | AW+Veh vs. Naive+Veh |  | >0.9999 | **ns** |
|  |  |  |  |  |  |  | AW+Tando vs. Naive+Veh |  | >0.9999 | **ns** |
| 1G |  | F (2, 12) = 6.018 | | 0.0155 | ***** |  | AW+Veh vs. AW+Tando | 5 | 0.0332 | ***** |
|  |  |  |  |  |  |  | AW+Veh vs. Naive+Veh |  | 0.0327 | ***** |
|  |  |  |  |  |  |  | AW+Tando vs. Naive+Veh |  | >0.9999 | **ns** |
| 1I |  | F (2, 12) = 7.227 | | 0.0087 | ****** |  | Naive+Veh vs. AW+Veh | 5 | 0.0153 | ***** |
|  |  |  |  |  |  |  | Naive+Veh vs. AW+Tando |  | >0.9999 | **ns** |
|  |  |  |  |  |  |  | AW+Veh vs. AW+Tando |  | 0.0251 | ***** |
| 1J |  | F (2, 12) = 8.036 | | 0.0061 | ****** |  | Naive+Veh vs. AW+Veh | 5 | 0.0485 | ***** |
|  |  |  |  |  |  |  | Naive+Veh vs. AW+Tando |  | 0.8916 | **ns** |
|  |  |  |  |  |  |  | AW+Veh vs. AW+Tando |  | 0.0065 | ****** |
| 2B | One-way ANOVA - repeated measured within subjects | F (3, 30) = 13.98 | | <0.0001 | ******** |  | Veh vs. 0.3 | 11 | >0.9999 | **ns** |
|  |  |  |  |  |  |  | Veh vs. 1 |  | 0.003 | ****** |
|  |  |  |  |  |  |  | Veh vs. 3 |  | <0.0001 | ******** |
| 2C |  | F (3, 30) = 5.745 | | P=0.0031 | ****** |  | Veh vs. 0.3 | 11 | >0.9999 | **ns** |
|  |  |  |  |  |  |  | Veh vs. 1 |  | >0.9999 | **ns** |
|  |  |  |  |  |  |  | Veh vs. 3 |  | 0.0073 | ****** |
| 2D |  | F (3, 27) = 2.863 | | P=0.0553 | **ns** |  | Veh vs. 0.3 | 10 | >0.9999 | **ns** |
|  |  |  |  |  |  |  | Veh vs. 1 |  | >0.9999 | **ns** |
|  |  |  |  |  |  |  | Veh vs. 3 |  | 0.0705 | **ns** |
| 2E | Two-way ANOVA | Interaction | F (34, 255) = 1.371 | 0.0909 | **ns** |  | 15 min: 1 vs. 3 | 6 | 0.0055 | ****** |
|  |  | Time | F (17, 255) = 58 | <0.0001 | ******** |  | 30 min: Veh vs. 1 |  | 0.0468 | ***** |
|  |  | Dose | F (2, 15) = 1.561 | P=0.2423 | **ns** |  | 40 min: Veh vs. 3 |  | 0.0087 | ****** |
|  |  | Subjects | F (15, 255) = 5.058 | P<0.0001 | ******** |  | 45 min: Veh vs. 3 |  | 0.0328 | ***** |

Table S1: Statistical analysis (continued)

| 2F | Two -tailed unpaired Student t test | - | 0.5825 | **ns** | - | - | 6 | - | **-** |
| --- | --- | --- | --- | --- | --- | --- | --- | --- | --- |
| 2G |  | - | 0.4139 | **ns** | - | - | 6 | - | **-** |
| 3B | One-way ANOVA | F (2, 86) = 7.575 | 0.0009 | ******* | Bonferroni | Naive+Veh vs. EtOH+Veh | 6 | 0.0072 | ****** |
|  |  |  |  |  |  | Naive+Veh vs. EtOH+Tando |  | >0.9999 | **ns** |
|  |  |  |  |  |  | EtOH+Veh vs. EtOH+Tando |  | 0.0018 | ****** |
| 3C | One-way ANOVA | F (2, 68) = 2 | 0.1432 | **ns** | Bonferroni | Naive+Veh vs. EtOH+Veh | 6 | 0.7348 | **ns** |
|  |  |  |  |  |  | Naive+Veh vs. EtOH+Tando |  | >0.9999 | **ns** |
|  |  |  |  |  |  | EtOH+Veh vs. EtOH+Tando |  | 0.1559 | **ns** |
| 3D | One-way ANOVA | F (2, 86) = 21.92 | <0.0001 | ******** |  | Naive+Veh vs. EtOH+Veh | 6 | <0.0001 | ******** |
|  |  |  |  |  |  | Naive+Veh vs. EtOH+Tando |  | >0.9999 | **ns** |
|  |  |  |  |  |  | EtOH+Veh vs. EtOH+Tando |  | <0.0001 | ******** |
| 4B | One-way ANOVA | F (2, 68) = 0.1004 | 0.9046 | **ns** |  | Naive+Veh vs. EtOH+Veh | 6 | >0.9999 | **ns** |
|  |  |  |  |  |  | Naive+Veh vs. EtOH+Tando |  | >0.9999 | **ns** |
|  |  |  |  |  |  | EtOH+Veh vs. EtOH+Tando |  | >0.9999 | **ns** |
| 4C | One-way ANOVA | F (2, 68) = 5.063 | 0.0089 | ****** |  | Naive+Veh vs. EtOH+Veh | 6 | 0.0473 | ***** |
|  |  |  |  |  |  | Naive+Veh vs. EtOH+Tando |  | >0.9999 | **ns** |
|  |  |  |  |  |  | EtOH+Veh vs. EtOH+Tando |  | 0.0145 | ***** |
| Fig. | Test | Chi-square, df, z | P value | Significance | Data analysed | Proliferation  (BrdU/KI67) | Differentiation  (BrdU/KI67/DCX) | | Total |
| 4C | Two-sided Chi-square | 6.972, 1, 2.641 | 0.0083 | ****** | AW + Tando | 42 | 87 | | **129** |
|  |  |  |  |  | AW + Veh | 57 | 59 | | **116** |
|  |  |  |  |  | Total | **99** | **146** | | **245** |
|  |  | 0.0008144, 1, 0.02854 | 0.9772 | **ns** | AW + Tando | 42 | 87 | | **129** |
|  |  |  |  |  | Naive + Veh | 53 | 109 | | **162** |
|  |  |  |  |  | Total | **95** | **196** | | **291** |
|  |  | 7.624, 1, 2.761 | 0.0058 | ****** | AW + Veh | 57 | 59 | | **116** |
|  |  |  |  |  | AW + Tando | 53 | 109 | | **162** |
|  |  |  |  |  | Total | **110** | **168** | | **278** |
